# Complement is a rat natural resistance factor to amoebic liver infection

**DOI:** 10.1042/BSR20180713

**Published:** 2018-10-02

**Authors:** Alfonso Olivos-García, Mario Nequiz, Scarlet Liceaga, Edith Mendoza, Porfirio Zúñiga, Azucena Cortes, Gabriel López-Velázquez, Sergio Enríquez-Flores, Emma Saavedra, Ruy Pérez-Tamayo

**Affiliations:** 1 Unidad de Investigación en Medicina Experimental, Facultad de Medicina, Universidad Nacional Autónoma de México, Ciudad de México, 04510, México; 2 Centro Médico Dalinde, Tuxpan 25, Roma sur, Ciudad de México, 06760, México; 3 Grupo de Estudio en Biomoléculas y Salud Infantil, Laboratorio de EIMyT, Instituto Nacional de Pediatría, Ciudad de México, 04530, México; 4 Departamento de Bioquímica, Instituto Nacional de Cardiología Ignacio Chávez, Ciudad de México, 14080, México

**Keywords:** complement, Entamoeba histolytica, innate immunity, resistance to amoebiasis

## Abstract

Amoebiasis is a parasitic disease caused by *Entamoeba histolytica*. This illness is prevalent in poor countries causing 100,000 deaths worldwide. Knowledge of the natural resistance mechanisms of rats to amoebic liver abscess (ALA) development may help to discover new pathogenic factors and to design novel therapeutic strategies against amoebiasis. In this work, histologic analyses suggested that the complement system may play a central role in rat natural resistance to ALA. *E. histolytica* trophozoites disappeared from rat liver within 6 h post-infection with minimal or no inflammatory infiltrate. *In vitro* findings indicate that rat complement was lethal for the parasite. Furthermore, hamsters became resistant to ALA by intravenous administration of fresh rat serum before infection. The amoebicidal potency of rat complement was 10 times higher than hamster complement and was not related to their respective CH50 levels. The alternative pathway of complement plays a central role in its toxicity to *E. histolytica* since trypan blue, which is a C3b receptor inhibitor, blocks its amoebicidal activity. These results suggest that amoebic membrane affinity, high for C3b and/or low for Factor H, in comparison with the hamster ones, may result in higher deposition of membrane complex attack on parasite surface and death.

## Introduction

*Entamoeba histolytica* is the protozoan parasite responsible for human amoebiasis that causes approximately 100,000 deaths each year worldwide [1]. It is an exclusive human disease and the parasite life cycle has not been reproduced in laboratory animals. However, experimental amoebic liver abscess (EALA) in hamsters is an extra intestinal model widely used to study this illness due to its reproducibility and similarity to the amoebic human liver disease that develop lethal lesions. Furthermore, some mouse strains are partially susceptible to acute EALA, in which tissue damage is repaired after parasite elimination observed from 3 to 7 days [2]. In contrast, rats are totally resistant to EALA [3]. Knowledge of the natural resistance mechanisms to EALA in rats may allow to stimulate a specific response in susceptible animals that could lead to new therapeutic strategies for human amoebiasis.

It has been suggested that during amoebic liver infection in mice, nitric oxide and hypochlorous acid derived from inflammatory cells may contribute to parasite elimination [4,5]. The clearance time of the parasite from liver of resistant animals could help to identify which cells and molecules are implicated in parasite elimination. The natural resistance mechanisms of rat to EALA have been scarcely analyzed. In this work, it was found that amoebae disappear from rat liver within 6 h post-infection, with minimal inflammatory infiltrate due to the powerful amoebicidal activity of its serum complement. Also, by comparing with either hamster or human (susceptible to ALA) C3 complement and inhibition of C3b receptor, it is suggested that alternative pathway of complement plays a central role in the potent rat complement toxicity to *E. histolytica* probably by a higher amoebic surface affinity for rat C3b and/or lower for Factor H (FH), which is a negative regulator of complement.

## Materials and methods

### Ethics statement

All experiments involving animals were performed in strict accordance with the Mexican Law for the Production, Care and Use of Laboratory Animals (NOM-062-ZOO-1999). All animal procedures were carried out under the protocol number 091–2016, approved by the Institutional Animal Care and Use Committees of the Facultad de Medicina, Universidad Nacional Autónoma de México. All efforts were made to minimize animal suffering.

### Parasites and virulence

Axenic cultures of virulent *E. histolytica* strain HM1-IMSS were maintained in TYI-S-33 medium according to standard protocols [6]. Virulence was defined as the ability of 1 × 10^6^ trophozoites to produce multiple liver abscesses in 4/4 hamsters (*Mesocricetus auratus*, 100 g weight) 7 days after intraportal inoculation. Such virulence was maintained by passing axenic trophozoites through hamster livers once a month, recovering the parasites from 7-day-old abscesses and again growing them axenically [7].

### Acute amoebic liver infection in hamsters and rats

Male or female hamsters (120 g) from our in-house colony were lightly anesthetized with barbital (6.3 mg/100 g body weight), the abdominal cavity was entered, and 1 × 10^6^ axenic trophozoites suspended in 0.2 ml of phosphate buffer saline (PBS) were intraportally injected as previously described [8]. Each experiment was performed in four animals. At different times after the intraportal injection, the animals were anesthetized with ether, killed by cardiac bleeding, the livers were removed, weighed, sliced for gross inspection, fixed in 3.7% formaldehyde in PBS and all liver lobes processed for histological study and stained with periodic acid–Schiff (PAS) technique. Amoebic infection in 200–250 g Wistar rats (*Rattus norvegicus albinus*) was made using the same procedure and injecting 1 × 10^6^/100 g body weight. The level of inflammatory response of each condition was determined by counting leukocytes present in 13 inflammatory foci.

### Serum from different animal species

Normal animals susceptible to amoebic liver infection (human and hamster), and resistant animals (Wistar rat, guinea pig, Long Evans rat, goat, lamb, cow, and rabbit) were bled (∼ 6 ml), their serum obtained at 4°C and stored at −70°C until used. For this analysis, four animals either male or female of each species were used.

### Complement CH50 activity by the hemolytic method

The serum complement activity was determined by the hemolytic method described by Morrison and Klein [9]. Briefly, sheep erythrocytes were sensitized (ES) with rabbit anti-sheep erythrocyte antibody prepared in our lab. To determine the reduction in the hemolytic complement titer, 200 μl of serially diluted serum in Veronal buffer (VB^++^) was incubated 30 min at 37°C after which 2.25 ml of 5 × 10^7^ ES/ml in VB^++^ was added and the incubation continued for an additional period of 15 min. Then, the samples were chilled at 4°C, centrifuged and the absorbance at 412 nm of the supernatant fractions indicated the percentage of hemolysis. The CH50 (U/ml) is defined as the amount of complement required for 50% lysis of the ES preparation.

### Complement CH50 activity by the liposome-based assay

The total CH50 complement activity in sera from different animal species was also determined using a commercial liposome immunoassay kit (Wako Autokit CH50). This method detects spectrophotometrically the G6PDH activity release after liposome lysis by the membrane attack complex of complement. The CH50 assay produces a calibration curve by plotting absorbance versus concentration [10]. It is important to mention that the CH50 interval of the liposome-based assay is reliable within 10–60 U/ml. Hence, assay results may not be accurate if samples are diluted. That is why, results outside this range are expressed as <10 and >60.

### Amoebic lytic effect of sera

Virulent amoebic trophozoites (1 × 10^6^) obtained from cell cultures were incubated with 1 ml of each serum for 2 h at 36°C. After this period, cell viability was determined by trypan blue exclusion.

### CVF purification

Cobra venom factor (CVF) was purified from crude commercial preparations of Naja haje venom (Sigma) by size exclusion and ion exchange chromatography [11]. Hypocomplementemic activity of purified CVF was evaluated *in vivo* by injecting 100 μg CVF intraperitoneally in a rat according to Van den Berg et al. [12]. After 1, 2, 3, and 4 days of CVF injection, blood (1.5 ml) from the tail vein of the rat was obtained and the *in vitro* amoebic lytic effect of sera was determined as mentioned above.

### *In vitro* and *in vivo* CVF effect

Fresh rat serum was decomplemented by CVF (5 μg/ml) incubation for 2 h at 36°C. The amoebic lytic complement effect was determined in fresh rat serum and in that incubated with CVF. Also, the CH50 level was determined in both sera by the hemolytic method as mentioned above. For *in vivo* experiments, CVF (100 μg) was injected intraperitoneally in four groups of rats (four/group) and 24 h later 1 × 10^6^ amoebae/100 g body weight were injected into the portal vein. In addition, four control groups of normal hamsters were intraportally injected with the same amount of parasites. The animals were killed at 6, 12, 24, and 48 h after parasite injection and their livers processed for histologic analysis, as described above. In addition, level of inflammatory response of each period was determined by counting leukocytes present in 13 inflammatory foci.

### Amoebic susceptibility to fresh rat serum diluted with hamster serum

Fresh rat serum was diluted with either fresh or heat decomplemented (56°C for 30 min) hamster serum in proportion 1:10, 1:1 and 10:1, and incubated 2 h at 36°C with virulent trophozoites of *E. histolytica* (1 × 10^6^/ml). Then amoebic viability was compared with the controls: (a) 100% fresh rat and hamster sera and (b) fresh rat serum decomplemented with CVF (5 μg/ml). This procedure was repeated three times in duplicates.

### Heterologous fresh serum in amoebic liver infection of hamster

Three milliliters of hamster blood was obtained from their cava vein and 3 ml of either fresh rat serum or inactivated by heating (56°C for 30 min) was immediately injected into the same vein, and 1 × 10^6^ amoebae were injected by intraportal route. The same procedure was performed in rats that received 5 ml of fresh hamster serum and 1 × 10^6^ amoebae/100 g body weight. Four animals per group were killed 7 days after parasite injection and the livers processed for histology as mentioned above.

### Amoebic resistance induction to fresh rat serum

Fresh rat serum was diluted in proportion 1:5 with PBS and sterilized by filtration with 0.45-μm millipore filters. Under sterile conditions, virulent trophozoites of *E. histolytica* (1 × 10^6^) were resuspended in 1 ml of diluted rat serum and incubated for 2 h at 36°C. Amoebic viability was determined by trypan blue exclusion. In a duplicate sample, the surviving parasites were cultivated and after 72 h were challenged again with diluted fresh rat serum. This procedure was repeated 10 times.

### C3 and C4 levels of rat, hamster, and human sera

The content of C3 and C4 in rat, hamster, and human fresh sera was quantified by the immunoturbidimetric specific protein method using the automatized equipment Architect c4000 of Abbott Laboratories, Diagnostics Division [13]. The protocol used goat anti-C3 and anti-C4 antibodies and both determinations were performed in three different animals and persons. Also, total protein in sera was determined.

### Effect of a C3b receptor inhibitor on amoebicidal activity of rat complement

To explore the possible participation of a C3b receptor of *E. histolytica* in the amoebicidal activity of rat complement, 0.5 × 10^6^ parasites were incubated for 1 h at room temperature under gentle mixing in tubes containing 5 mg trypan blue (a C3b receptor inhibitor [14]) dissolved in 0.5 ml of TYI-S-33 medium. After that, the cells were collected by centrifuging at 500 ***g*** for 3 min. Then 0.45 ml of the supernatant was discarded and 0.45 ml of fresh rat serum previously incubated with 10 mg trypan blue for 1 h at 4°C was added. The cells were incubated for 2 h at 37°C in a rocker and viability was determined as mentioned above. For this experiment, sera from three males and two females Wistar rats were used. Viability was also compared with duplicated parasite samples (control) that contained culture medium instead of sera.

### Statistical analysis

When indicated, statistical test used is mentioned in the figure legend and was performed using GraphPad Prism version 5.00 for Windows (GraphPad Software, San Diego,CA, U.S.A.).

## Results

### *E. histolytica* disappears from rat liver before 6 h

**Figure 1:**
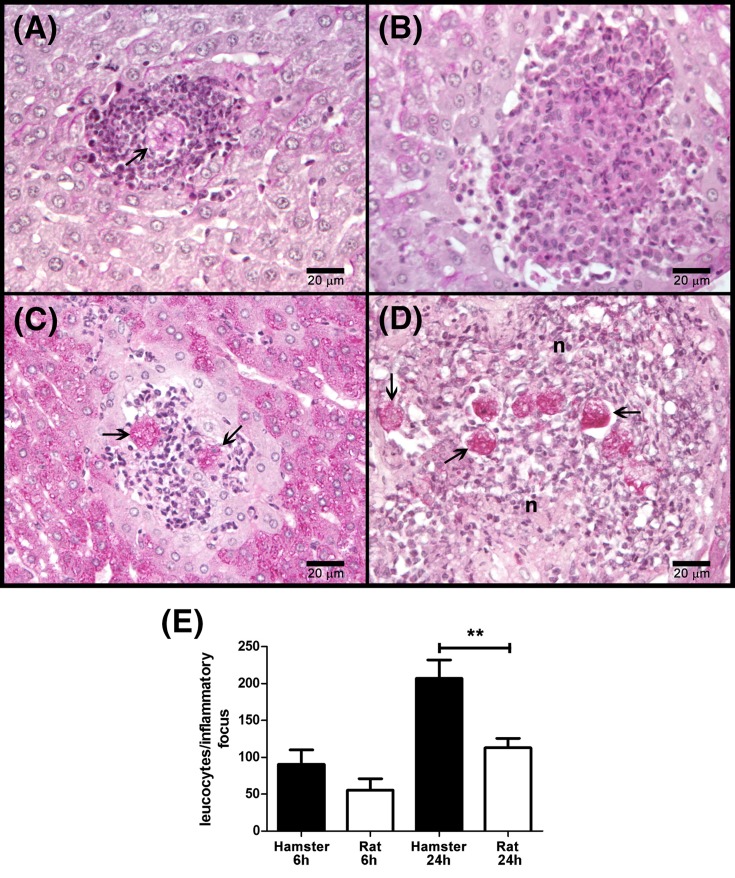
*E. histolytica* survival in hamster and rat livers Amoebae (1 × 10^6^/100 g body weight) were injected into the portal vein of normal hamsters and rats (four/group) and after 6 and 24 h the slices of fixed livers were stained with PAS. The inflammatory response was also determined by counting leukocytes present in inflammatory foci. In both rat (**A**) and hamster (**C**), after 6 h of infection, well-preserved amoebae are surrounded by few inflammatory cells without tissue damage. After 24 h, in hamster liver (**D**) abundant well-preserved parasites are contained in inflammatory foci with tissue destruction, whereas only residual inflammatory lesions without amoebae are observed in the rat liver (**B**). Amoebae (arrows) and necrosis (n) are indicated. The inflammatory level of livers (**E**) is highest only in hamsters after 24 of infection. We used one-way ANOVA with Bonferroni’s correction to compare specific groups (significance level set at *P*≤0.0083). Asterisks indicate significant differences between groups as indicated; **P≤0.01. Data are presented as mean ± SEM.

To determine the clearance time of the parasite, a time course of amoebic liver infection was analyzed in rats and hamsters. The clearance time of the parasite in rat liver was 6 h, with minimal inflammatory infiltrate (Figure 1A,E). Also after 24 h, only residual lesions without parasites were observed (Figure 1B,E). This is in contrast with what occurs in hamster that shows gradual increase in amoebic proliferation, inflammatory infiltrate, and tissue damage (Figure 1C–E).

### Total *in vitro* amoebicidal activity of rat serum correlates with its resistance to amoebic liver infection

**Figure 2:**
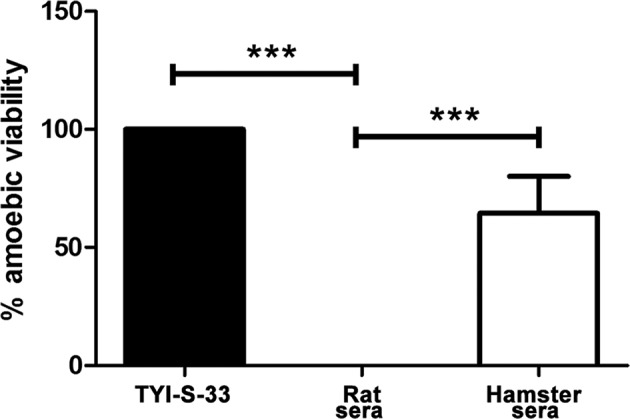
*E. histolytica* susceptibility to hamster and rat fresh sera Amoebae (1 × 10^6^/ml) were incubated with fresh hamster or rat sera (four animals/species) either male or female and after 2 h the viability was determined. Statistical analysis was done by using one-way ANOVA, the comparison between the groups was done according Bonferroni’s correction (significance level, *P*≤0.016). Asterisks indicate significant differences between groups as indicated; ***P≤0.001. Data are presented as mean ± SEM.

To explore if complement was related to the faster amoebic clearance from rat liver, the *in vitro* amoebicidal activity of rat (resistant) and hamster (susceptible) fresh sera was determined. While the amoebicidal activity of hamster serum from different animals is variable (26–96%), which derived from rats is 100% lytic for the parasite and does not show intraespecies changes (Figure 2).

### CH50 complement level of different animal species is not related to their amoebicidal activity

**Figure 3:**
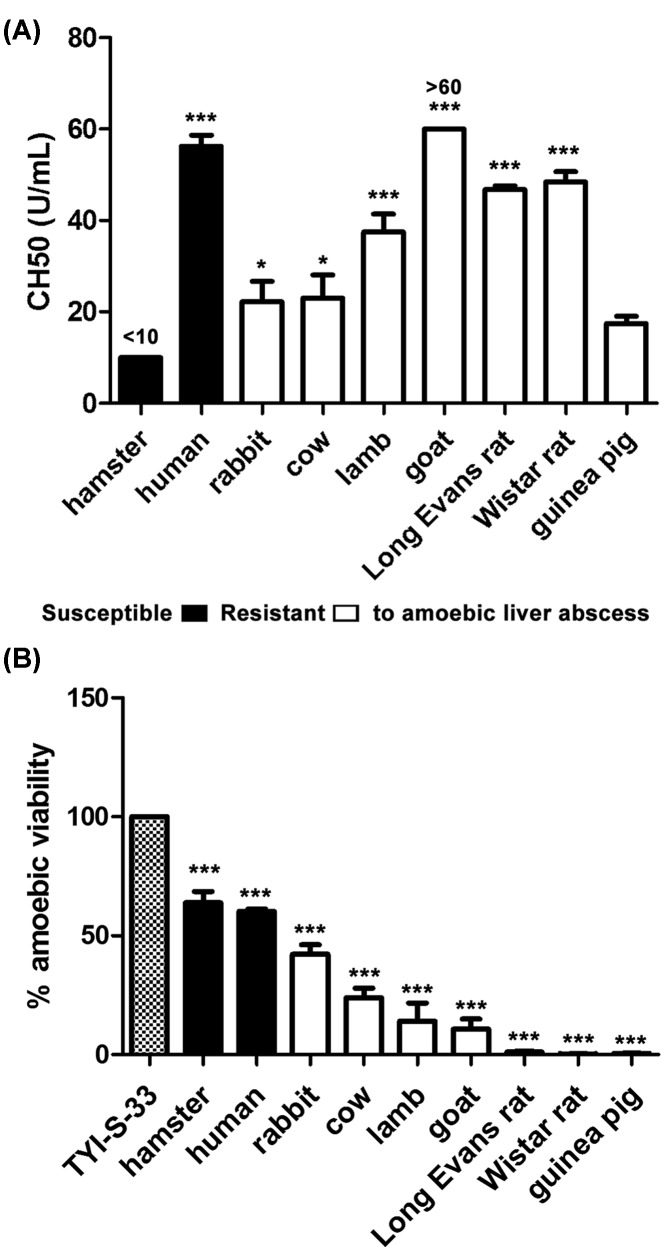
CH50 levels of sera from different mammals and their correlation with amoebicidal activity (**A**) CH50 activity of fresh sera from different animal species (four/group) was determined by a liposome-based assay. From all animals, only hamster and goat were not within the reliable range of this assay (10–60 U/ml). (**B**) *E. histolytica* parasites (1 × 10^6^/ml) were incubated with fresh sera from different animal species (four/species) and after 2 h viability was determined. Statistical analysis was done by using one-way ANOVA with Dunett post-tests (*α* set at 5%). Asterisks indicate significant differences respect to the hamster (in A) and control TYI-S-33 medium (in B) groups as indicated; *P≤0.05, ***P≤0.001. Data are presented as mean ± SEM.

The CH50 level of serum complement of normal animals either susceptible (hamster, human) or resistant (rabbit, cow, lamb, goat, Long Evans and Wistar rats and guinea pig) to amoebiasis was determined and compared with their amoebicidal activity. From all the analyzed animal types, goat and human showed the highest CH50 levels (all goat samples >60 and human from 50 to >60). Also, the hamster had the lowest CH50 level (all samples <10) and the other animals showed variable levels from 17.5 to 48.5 (Figure 3A). However, the CH50 complement level of the different animals did not correlate with their amoebicidal activity since humans and rats that had a higher CH50 activity allowed amoebic survival of 60 and 0%, respectively. In addition, hamster sera with the lowest CH50 level showed amoebicidal activity similar to the human group (Figure 3B).

### The amoebicidal activity of rat serum is higher than hamster serum one and is due to complement

**Figure 4:**
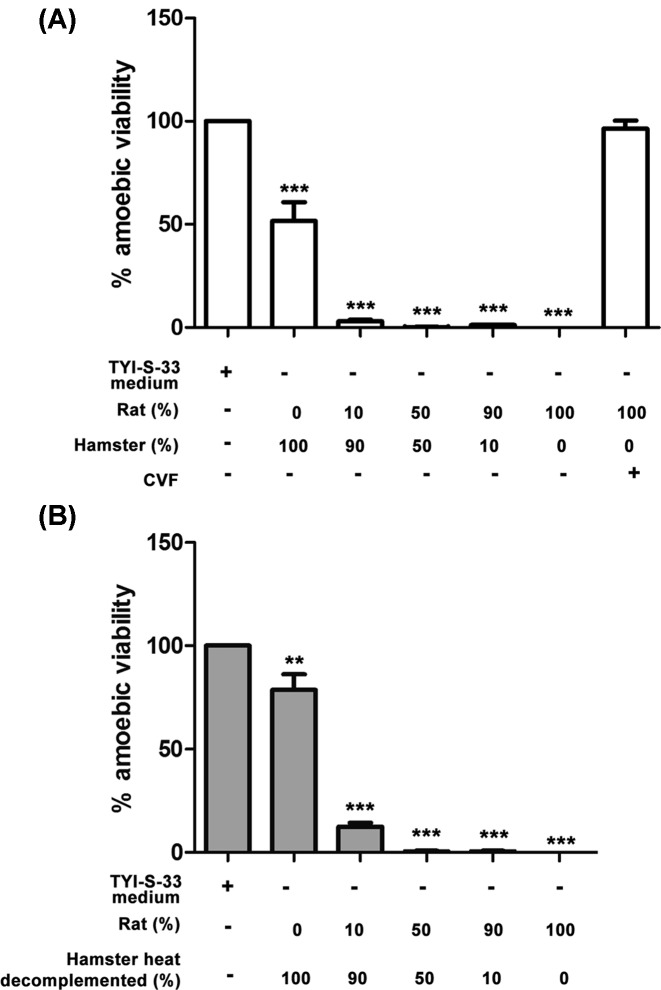
Amoebicidal activity or fresh rat serum diluted with hamster serum (**A**) *E. histolytica* (1 × 10^6^/ml) was exposed to rat fresh serum either preincubated with CVF or diluted with fresh hamster serum and after 2 h of incubation cell viability was determined. (**B**) A similar procedure was performed with amoebae incubated with fresh rat serum diluted with hamster serum previously decomplemented by heat. Determinations in (A) and (B) were performed three times in duplicates. Statistical analysis was done by using one-way ANOVA with Dunett post-tests (*α* set at 5%). Asterisks indicate significant differences respect to the control (amoebae in TYI-S-33 medium) as indicated; **P≤0.01, ***P≤0.001. Data are presented as mean ± SEM.

The total amoebicidal activity of rat serum is unchanged if it is diluted 10-fold with fresh hamster sera. Such powerful effect is due to complement since it was totally inhibited with CVF that specifically consumes complement due to its C3 convertase activity (Figure 4A). In addition, when diluted fresh rat serum with heat inactivated hamster serum (hypocomplementemic), rat complement maintains its total amoebicidal activity in 2-fold dilution and even when diluted by 10-fold it kills almost all parasites (87%) (Figure 4B). Another interesting result was that amoebae did not develop resistance to rat complement by repeated exposures (10 times) to LD50 concentrations.

### Complement is a rat natural resistance factor to amoebic liver infection

**Figure 5:**
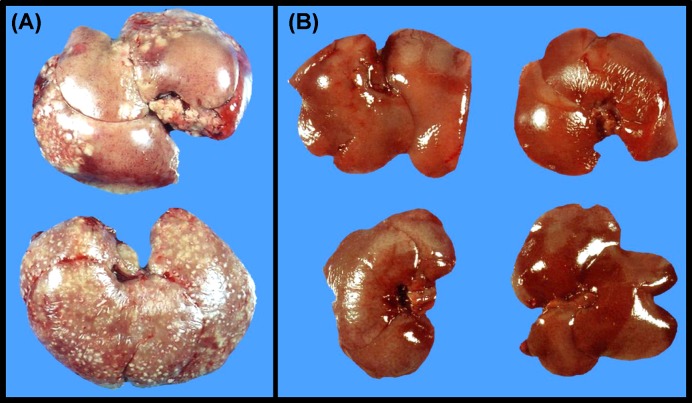
Hamster resistance to amoebic liver infection induced by intravenous transference of fresh rat serum Three milliliters of hamster blood was obtained from their cava vein and 3 ml of either fresh rat serum or inactivated by heating (controls) was immediately injected into the same vein, and 1 × 10^6^ amoebae were injected by intraportal route (four animals/group). After 7 days, control livers (**A**) show hepatomegaly and multiple tissue lesions whereas fresh rat serum transference completely inhibited liver abscess (**B**).

To explore if complement is involved in the rat natural resistance to amoebic liver infection, the blood of live hamster was diluted with either fresh rat serum or decomplemented by heating before liver infection with the amoebae. From these treatments, only fresh rat serum conferred hamster resistance to amoebic liver infection. Also, in contrast with control hamsters (Figure 5A), the parasites disappeared from livers in the absence of tissue damage (Figure 5B).

### Complement is not the only rat resistance factor to amoebic liver infection

**Figure 6:**
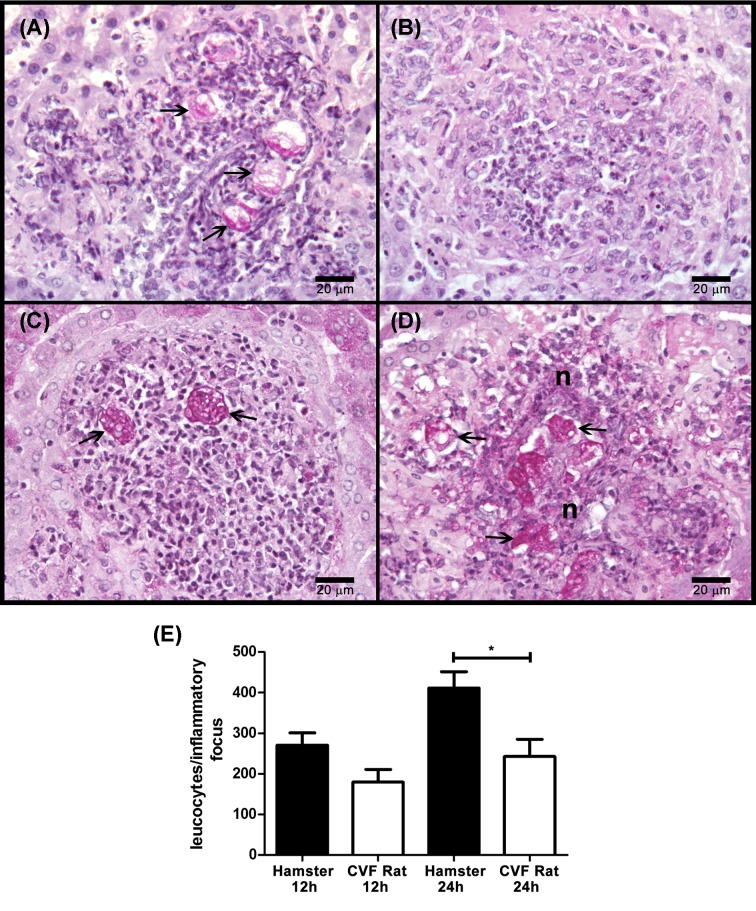
*E. histolytica* survival in normal hamster and hypocomplementemic rat livers Parasites (1 × 10^6^/100 g body weight) were injected into the portal vein of a group of normal hamsters and rats decomplemented with CVF (four/group). The inflammatory response was also determined by counting leukocytes present in inflammatory foci. After 12 and 24 h, their livers were processed for histology and PAS staining. In both rat (**A**) and hamster (**C**), after 12 h of infection well-preserved amoebae are surrounded by few inflammatory cells without tissue damage. After 24 h, in hamster liver (**D**) abundant well-preserved parasites are contained in inflammatory foci with tissue destruction whereas in the decomplemented rat (**B**) only residual inflammatory lesions without amoebae are observed. Amoebae (arrows) and necrosis (n) are indicated. The inflammatory level of livers (**E**) is highest only in hamster after 24 h of infection. Statistical analysis was done with one-way ANOVA, *α* was set at 0.0125 according with Bonferroni’s correction. Asterisks indicate significant differences between groups as indicated; *P≤0.05. Data are presented as mean ± SEM.

To determine if complement is the only resistant factor of the rat to prevent amoebic liver infection, parasites were injected into the portal vein of rats decomplemented with CVF and the evolution of the liver infection was analyzed by histology and compared with amoebic liver infection in hamsters. Hypocomplementemia did not induce rat susceptibility to amoebic liver infection. Instead, it only prolonged amoebic survival up to 12 h, in which parasites were surrounded by leukocytes (Figure 6A) before they disappear at 24 h (Figure 6B). In contrast, the hamster liver was characterized by gradual increase in amoebae, inflammatory infiltrated and tissue damage (Figure 6C–E). In addition, the CVF used in our experiments showed good efficacy since it was able to reduce the CH50 level of the rat serum <10 after 1, 2, and 3 days of injection and the same sera allowed amoebic survival of 98, 97, and 88% respectively, which is in contrast with fresh rat serum that was 100% toxic on the parasite.

### A C3b receptor is related to the potent amoebicidal activity of rat complement

**Figure 7:**
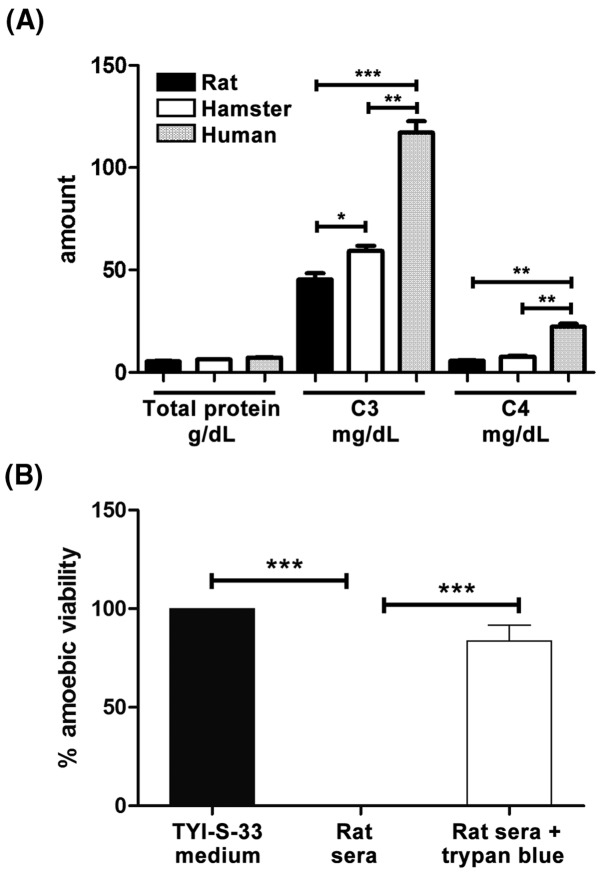
C3 and C4 sera levels and effect of trypan blue on the amoebicidal activity of rat complement (**A**) C3 and C4 level were determined by an immunoturbidimetric method in sera from three different resistant (rat) and susceptible (hamster and human) species to amoebic liver infection. Also, total protein was determined in all samples. (**B**) Fresh sera from five different rats either male or female were incubated for 2 h with or without trypan blue (an inhibitor of C3b receptor) and parasite viability determined and compared with amoebae viability in culture medium (TYI-S-33). Statistical analysis was done with one-way ANOVA, *α* was set at 0.016 according with Bonferroni’s correction. Asterisks indicate significant differences between groups as indicated; *P≤0.05, **P≤0.01 ***P≤0.001. Data are presented as mean ± SEM.

To determine if C3 and C4 levels were associated with the amoebicidal activity of rat complement, the C3 and C4 content of rat serum was compared with the hamster and human sera that show similar toxicity levels against *E. histolytica*. Rat showed the lowest levels of C3 and C4 in comparison with hamster and human that are susceptible to amoebic liver infection (Figure 7A). On the other hand, it was investigated if an amoebic C3b receptor could be involved in the amoebicidal activity of rat complement. For that purpose, amoebae were incubated with fresh rat serum in the presence of trypan blue, which binds to the C3b receptor in many cells from different species [14]. In the amoebic cultures, trypan blue did not show toxicity on *E. histolytica*; however, it was able to considerably inhibit the amoebicidal activity of rat complement (Figure 7B) suggesting that an amoebic C3b receptor is involved in this phenomenon.

## Discussion

Parasites have evolved many strategies to survive in their hosts mostly by avoiding innate (complement and TLR recognition) and adaptive (immunomodulation) immunity [15–18]. The presence, absence, and/or structural differences of molecules involved in these immune host responses among species could determine the susceptibility or resistance to a specific parasite infection.

In the experiments performed here, all animal species examined showed variable serum CH50 levels (Figure 3A) that failed to correlate with their amoebicidal effect (Figure 3B). Human complement activation by classical and alternative pathways has been observed in *E. histolytica* [19,20]. The alternative pathway or mannose-complement pathways involved in the amoebicidal sera effect could not be ruled out since the CH50 assays used in this work detect only the classical pathway [9,10] and species-specific recognition by the alternative pathway of complement has been documented [21]. The total amoebic lytic effect, only shown by fresh sera from guinea pig and rats (Wistar and L. Evans strains), was striking. Resistance to human complement observed in different amoebic strains [22,23] or by induction with repeated challenges to either human or hamster fresh sera [24,25] correlates with *E. histolytica* virulence. In agreement, from all animal species analyzed virulent amoebae showed the highest resistance level to hamster and human fresh sera (Figure 3B). Amoebic adhesin containing an epitope of CD59 (MAC-inhibitory protein) [26], complement activation in fluid phase by an amoebic cysteine protease [20] and uptake-exposure of complement regulators to membrane [27] are involved in amoebic resistance to human complement. In contrast, in the experiments shown here, virulent *E. histolytica* was completely susceptible to fresh rat serum (Figure 2) and did not develop resistance after 10 repeated exposures to sublethal rat serum dilution. The potent amoebicidal effect of fresh rat serum is maintained even when diluted with fresh or decomplemented hamster serum (Figure 4). Such effect was demonstrated to be complement, since ∼100% parasite viability was observed with rat serum preincubated with CVF (Figure 4A) that specifically consumes complement due to its C3 convertase activity. Although C3 is a complement protein present in all three pathways and C4 belong to the classical pathway [28], their content in serum was not related with the amoebicidal activity of sera from resistant and susceptible species to amoebic liver infection (Figure 7A).

On the other hand, it is known that MAC-dependent cell lysis is up to 100-fold more efficient on surfaces containing bound C3b than on surfaces devoid of C3b [29]. Moreover, properdin binding to the complement activating surface is necessary to stabilize the alternative pathway C3 convertase. Furthermore, it has been observed that activation of the rat alternative complement pathway by *Babesia rodhaini* results in binding of C3b via C3b receptor on the parasite. Such binding facilitates erythrocytes parasite infection that contains a C3b receptor too and could be inhibited by trypan blue [30]. It is well documented that trypan blue is able to block C3b complement receptors present in different parasites and cells from different animal species [14,31–33]. Similarly, binding of rat complement C3b to the amoebic surface may be involved in the potent amoebicidal activity of rat complement, since its effect was inhibited by trypan blue (Figure 7B), which is a blocker of the C3b receptors. [14]. Factor H (FH) is a key soluble negative regulator of the alternative pathway that promotes inactive C3b formation and operates in both the fluid phase and on self surfaces. Several pathogens like *Borrelia hermsii, Neisseria meningitidis*, group A *streptococci, Yersinia enterocolitica*, and *Candida albicans* recruit FH to regulate complement activation on their surfaces [34,35]. On the contrary, low affinity of amoebic surface for FH may be involved in the powerful activity of the rat complement. Our findings point out to the alternative pathway of rat complement as the principal responsible for its powerful amoebicidal activity; currently we are studying if the amebic surface affinity for C3b (high) and FH (low) are involved.

In several animals like mouse, rabbit and guinea pig, the parasite can induce reversible acute amoebic liver tissue damage and amoebae are eliminated at different times [36,4]. In contrast, the amoebic liver infection in gerbils and hamsters is lethal as in humans [2]. Therefore, it may be hypothesized that all animal species mentioned above may have differences in their innate and/or acquired immunity that may promote or avoid amoebic survival and tissue damage. In the *in vivo* experiments reported here, *E. histolytica* disappeared from rat liver before 6 h with minimal or no inflammatory infiltrate (Figure 1) and hypocomplementemia prolonged amoebic clearance time to 12 h in which some parasites were observed surrounded by inflammatory cells (Figure 6). This suggests that complement was responsible for early amoebic elimination (before 6 h) from the rat liver. Also, it supports the idea that in this animal, and in contrast with hamsters, inflammatory cells could be responsible for the late amoebic disappearance (after 12 h) probably by the activity of iNOS enzyme that produces nitric oxide, a free radical extremely toxic for *E. histolytica* [37]. In agreement, macrophages of different species have great differences in iNOS expression to a common stimulus; for example, alveolar macrophages from rat, but not hamster or human, overexpress iNOS protein when challenge with LPS plus IFN-γ [38,39].

On the other hand, when fresh rat serum was injected to hamsters by intracava vein injection followed by parasite inoculation through the portal vein, no tissue damage was observed after 7 days (Figure 5). Then by exploring the early stages of infection histologic similarities with rat liver infection were observed i.e*.* amoebae up to 6 h were surrounded by minimal or no inflammatory infiltrate (Figure 1). Also, no protective effect was observed when fresh rat serum inactivated by heating at 56°C for 30 min was used. These findings mean that hamsters become resistant to amoebic liver infection (like rats) even in the presence of infection-permissive cells and molecules of the hamster. On the contrary, fresh hamster serum injected into the rat cava vein did not modify rat amoebic liver resistance, possibly through constant complement synthesis and the presence of reactive macrophages.

In summary, these results show that complement levels (CH50) present in fresh sera of different animal species do not correlate with their *in vitro* amoebic lytic capacity. Also, serum complement, through the alternative pathway, contributes to the natural resistance of rat to experimental liver amoebiasis. Of course, rat may harbor other resistance factors (such as macrophages) for resistance to *E. histolytica* infection.

## Abbreviations

ALA: amoebic liver abscess
CVF: cobra venom factor
EALA: experimental amoebic liver abscess
iNOS: nitric oxide synthase
IFN-γ: interferon gamma
